# Time-Restricted Eating Improves Quality of Life Measures in Overweight Humans

**DOI:** 10.3390/nu13051430

**Published:** 2021-04-23

**Authors:** Ainslee Crose, Alison Alvear, Stephanie Singroy, Qi Wang, Emily Manoogian, Satchidananda Panda, Douglas G. Mashek, Lisa S. Chow

**Affiliations:** 1 Division of Diabetes, Endocrinology and Metabolism, University of Minnesota, Minneapolis, MN 55455, USA; neuxx050@umn.edu (A.C.); bunio001@umn.edu (A.A.); ssingroy@umn.edu (S.S.); dmashek@umn.edu (D.G.M.); 2 Clinical and Translational Science Institute, University of Minnesota, Minneapolis, MN 55455, USA; wangx890@umn.edu; 3 Salk Institute for Biological Studies, La Jolla, San Diego, CA 92037, USA; emily.manoogian@gmail.com (E.M.); panda@salk.edu (S.P.); 4 Department of Biochemistry, Molecular Biology and Biophysics, University of Minnesota, Minneapolis, MN 55455, USA

**Keywords:** time-restricted eating, quality of life, SF-36

## Abstract

Time-restricted eating (TRE) reduces weight in humans, but its effects on quality of life have not been well characterized. By performing a secondary analysis of a randomized clinical trial, we examined the effects of TRE (12-week intervention, 8 h eating window) vs. non-TRE (unrestricted eating) on quality of life (QoL) measures. Twenty subjects with overweight and prolonged eating window (mean (SD): 15.4 h (0.9)) were randomized to either 12 weeks of TRE (8 h eating window: (*n* = 11)) or non-TRE (*n* = 9). QoL data were collected with the 36-item Short Form Survey (SF-36) pre- and post-intervention. Given a two-way ANOVA model and post-hoc *t*-test analysis, the TRE group improved limitations due to emotional health post-intervention: +97.0 (10.0)) vs. baseline: +66.7 (42.2) (*p* = 0.02) and perceived change in health over the last year end intervention: +68.2 (16.2) vs. baseline: +52.3 (23.6) (*p* = 0.001) relative to baseline. The TRE group improved limitations due to emotional health TRE: +97.0 (10.0) vs. non-TRE: +55.6 (44.1) (*p* = 0.05) and perceived change in health (TRE: +68.2 (16.2) vs. non-TRE: +44.4 (31.6) (*p* = 0.04) relative to the non-TRE group at post-intervention (*p* < 0.025). In conclusion, 12 weeks of TRE does not adversely affect QoL and may be associated with modest improvements in QoL relative to baseline and unrestricted eating; these findings support future studies examining TRE compliance and durability.

## 1. Introduction

The obesity epidemic is well established [1,2]. Thirty-eight percent of American adults are obese [3], yet studies have shown only a small percentage of adults who attempt weight loss, 20–31.3%, are able to maintain it [4,5,6]. This struggle with weight loss is likely due to a number of factors, including metabolic adaptations and the difficulty of adhering to traditional dietary interventions such as calorie restriction [7].

Time-restricted eating (TRE) is emerging as a potential treatment option for weight loss and improved metabolic health [8,9]. A pilot study of 8 h TRE showed a 2.6% decrease in body weight after a 12-week trial period [9]. Another recent study which compared 4 h and 6 h TRE for 8 weeks found a similar weight loss of ~3% in both groups [10]. TRE has metabolic benefits in addition to, and possibly separate from, weight loss. A 2018 study by Sutton et al. showed improvements in insulin sensitivity and blood pressure in men with pre-diabetes even in the absence of weight loss [11]. A recent study of individuals with metabolic syndrome found improvements in weight, blood pressure, and lipids with 10 h TRE after 12 weeks, but they did not find a correlation between weight loss and these metabolic improvements, also indicating TRE may have metabolic benefits separate from weight loss [12].

As TRE has increased in popularity in the last decade, its ability to maintain long-term weight loss has not been studied, though it has been hypothesized that the simplified approach of TRE may be easier to sustain than traditional calorie restriction as it is a simplified way of eating that allows for ad libitum intake during a specific time window and does not require time-consuming calorie counting [13]. If TRE and associated weight loss can be demonstrated to improve quality of life (QoL), presumably this would facilitate adherence, though the relationship between weight loss and QoL is unclear. A systematic review looking at health-related QoL after weight loss found improvement in some trials but not others [14].

Recently, a small non-randomized study of TRE in ten older adults did not find a negative effect on QoL [15]. A recent non-randomized study of 32 women with obesity found a statistically significant improvement in QoL post-TRE as measured by the WHOQoL survey [16]. A larger secondary analysis of 99 non-randomized participants who participated in TRE found a significant improvement in health related QoL (HRQoL) as measured by the EQ-5D VAS [17]. However, to our knowledge, there have not been any studies assessing QoL with the SF-36, a well-validated and widely used QoL survey that offers insight into different components of QoL, both physical and mental. The specific goal of this project was to evaluate the effect of TRE vs. non-TRE on QoL as measured by the SF-36. We hypothesized that in the setting of a randomized intervention, TRE will improve QoL relative to baseline and relative to non-TRE.

## 2. Materials and Methods

### 2.1. Study Design

This is a secondary analysis of our previously published study that was a randomized, controlled trial comparing TRE with unrestricted eating (non-TRE) [8]. In brief, 20 overweight subjects (17F/3M, mean (SD), age: 45.5 years (12.1), BMI: 34.1 kg/m^2^ (7.5)) with a prolonged eating window (15.4 h (0.9)) were randomized to either 12 weeks of TRE (*n* = 11) or non-TRE (*n* = 9). Participants randomized to the TRE group were instructed to eat without restrictions during their self-selected 8 h eating window. Outside the eating window, the TRE group participants were only allowed water and medications. Participants in both groups logged all food and drink on the myCircadianClock app throughout baseline and intervention. QoL data were collected using the 36-item Short Form Survey (SF-36) at baseline and at post-intervention. The University of Minnesota’s Institutional Review Board approved the protocol. The Salk Institute’s IRB approved use of the myCircadianClock app for this study. All participants provided written informed consent before participation. The study was registered at Clinicaltrials.gov NCT03129581, accessed 26 April 2017.

### 2.2. SF-36

Given that we were studying healthy humans who were overweight, we used the SF-36 survey to assess self-described QoL at baseline and at post-intervention. The SF-36 was developed initially from the Medical Outcomes Study in 1992 by John Ware [18]. Version 2 was released in 1996 [19]. The SF-36 is one of the most well-established, reliable, and validated QoL surveys [18,20,21]. A 1992 study by Brazier et al. showed that the internal consistency, measured by Cronbach’s alpha, of each of the eight scales ranged from 0.73 to 0.96 and the test–retest reliability from 0.6 to 0.81 [22]. In contrast to disease-specific health-related QoL surveys, the SF-36 permits comparison with the general population [18]. An additional advantage of the SF-36 is its ease of use, with typical completion within 5–10 min [23].

The survey measures QoL from many perspectives. Eight scales including physical functioning, role limitations due to physical health, bodily pain, general health perceptions, energy/fatigue (also called vitality), social functioning, general mental health (emotional well-being), and role limitations due to emotional health are measured by the results of 36 questions, with an additional question on health change/transition. The 36 questions contained answer choices in the form of Likert scales or yes/no responses that asked participants to consider their health in the last four weeks. All scores were recoded with 100 as a maximum score; a higher score indicates a better QoL. All eight scales are valid measures of either physical health, mental health, or both. Physical functioning, role limitations due to physical health, and bodily pain are valid measures of the physical component of health [18]. General mental health or emotional well-being, role limitations due to emotional health, and social functioning are valid measures of the mental component of health. Energy and general health are valid measures of both components. The scales that contribute more to the physical component of health are more responsive to interventions for physical morbidity, and the same is true for mental health [24]. Appendix B reports the scoring and utilized questions for each scale.

Unlike the eight health scales, the health transition question, which compares current health relative to health one year ago, has not been as extensively validated, and its consistency over time remains less well established [25]. When assessing criterion validity against the General Health Ratings Index, a correlation was found between scores on the transition question and ratings of general health, with those who evaluated their health as “much better” on the SF-36 improving by 13.2 points on the GHRI, and those that rated their health as “somewhat better” improving by 5.8 points [24]. While there is no inter-item consistency for the transition question, which reduces its utility in describing changes at the individual level, it is useful in describing clinical change at the group level [25].

### 2.3. Statistical Analysis

Treatment groups were compared pre-intervention and post-intervention for each scale and the transition question using a two-way ANOVA model which included the group effect, time effect, and group-by-time interaction. For QoL measures in which the group-by-time interaction was significant, post-hoc *t*-tests were done to determine whether the pre–post change was significant in the TRE or non-TRE group. To adjust for multiple comparisons, a *p*-value of 0.025 (0.05/2) was used for significance for post-hoc *t*-tests. To determine whether there was a correlation between the magnitude of change in the eating window and the magnitude in change of scales, Pearson correlation was measured. All analysis was performed in SAS (v 9.3) with *p* < 0.05 considered statistically significant.

## 3. Results

### 3.1. TRE and Weight Loss

As previously described [8], twenty participants (17 women and 3 men; mean (SD): age 45.5 (12.1) years, BMI 34.1 (7.5) completed the study. The baseline characteristics of the study participants (Table 1) have been previously reported and are summarized here for convenience. The prescribed intervention eating window was eight hours, but due to adherence, the average at the end of the intervention was 9.9 (2.0) hours and the average in the non-intervention group was 15.1 h (1.1) (*p* < 0.01) [8].

### 3.2. SF-36 Measures

Pre-intervention, there were no significant differences between the TRE and non-TRE groups. Using the two-way ANOVA model, there was found to be a significant group-by-time interaction (Table 2) for the role limitations due to emotional health (*p* = 0.04) and health transition (*p* = 0.05). Post-hoc *t*-tests (Table 3) showed a significant improvement in both variables within the TRE group (*p* < 0.025 for both variables) and no significant change within the non-TRE group. Participants in the TRE group did not report any significant alteration of physical functioning, role limitations due to physical functioning, bodily pain, general health, energy, social functioning, or emotional well-being.

Results are reported as (mean (standard deviation) (range of participant scores)). The minimum score for each scale is 0, and the maximum is 100.

### 3.3. Correlation between SF-36 Results and Change in Eating Window and Weight Loss

As previously reported [8], the TRE group reduced body weight by 3.7% [1.8%] from an average of 95.2 (22.6) kg to 91.6 (21.5) kg (*p* < 0.01). Weight change in the non-intervention group was not significant, with a change from 100.9 (28.1) kg to 99.4 (28.1) kg (*p* = 0.09). There was no correlation between changes in the SF-36 response and extent of weight loss. There was also no correlation between the changes in the SF-36 response and the extent of eating window restriction (Appendix A).

## 4. Discussion

Using the SF-36 survey, we found that 12 weeks of TRE did not adversely affect QoL and may be associated with modest improvement in several QoL measures relative to baseline and unrestricted eating. Specifically, TRE did not negatively impact the remaining SF-36 measures, including physical functioning, role limitations due to physical functioning, emotional well-being, bodily pain, energy, social functioning, or general health relative to non-TRE or baseline. QoL was improved as shown by the health transition score, described as “perception of health change in the past year”, and “role limitations due to emotional health”. The benefits of TRE on QoL appeared to be related to the TRE intervention, as the changes in QoL were independent of the change in in weight or eating window, supporting acceptability of the TRE intervention.

Although TRE has been shown to reduce weight [8,15,26] and improve metabolic measures [10,27], only a limited number of studies have evaluated its effects on QoL, and none have done so using the SF-36. Most of these studies have shown that TRE has an either neutral or positive effect on QoL [15,16,17,28]. A 2020 non-randomized study of 14 individuals with Type 2 diabetes reported that TRE (~9 h window for 4 weeks) was not associated with any significant changes in aspects of QoL as measured by the AQoL-8D [28]. A 2019 non-randomized study by Anton et al. found that older adults (≥age 65) who performed TRE (~8 h window for 4 weeks) experienced a non-statistically significant improvement of 5–8% on mental and physical health domains as measured by SF-12, with the largest increase in the physical health domain [15]. A 2020 non-randomized study by Kesztyus et al. used the EQ-5D VAS to measure HRQoL pre- and post-TRE (~8–9 h window for 3 months) [17]. They found a significant improvement in HRQoL relative to baseline in the TRE group, independent of weight loss, which is similar to our finding using the SF-36, though the EQ-5D VAS does not look at different aspects of HRQoL but rather a single overall measure. Our results using the SF-36 provide a more nuanced view of the specific impact of TRE on multiple aspects of QoL. Recently, Schroder et al. showed in a study of 32 women with obesity (TRE: 8 h for three months) a statistically significant improvement in QoL as measured by the WHO-QoL validated for Brazil. This improvement was seen in the TRE group but not the control group [16]. Specifically, they found an improvement in the self-perception domain of QoL, which may be attributed to improved body image related to weight loss, though they did not examine whether there was a correlation between those variables. Our findings in men and women with overweight build on this study’s findings of women with obesity to show improved QoL after a TRE intervention. Another study by Antoni et al. in 2018 (TRE: delayed breakfast by 1.5 h and advanced dinner by 1.5 h) did not directly measure QoL but did administer an exit survey to participants after the 10-week period of reducing eating window by 4–5 h, where 4 out of 11 of whom indicated that TRE negatively affected opportunities for social eating and drinking [29]. Our study complements and extends a previous study of 11 males who restricted their eating window to 8 h per day for 5 days that included qualitative interviews in which participants reported a mostly positive view of TRE and its associated structure, describing increased energy and feelings of well-being, while noting potential barriers to adherence including work/family schedules and social life [30]. While we did not find a similar negative effect on social functioning, further investigation is warranted.

When evaluating the improvements in QoL from TRE, the confounding variable of weight loss must be considered. As the TRE group lost more weight than the non-TRE group, it is possible that the improvements in QoL may be due to weight loss rather than the TRE intervention, though we did not see a significant correlation between the amount of weight loss and improvement in SF-36 measures. According to a systemic review by Kolotkin et al., the literature has not reported a consistent association between weight loss and improved QoL even when limiting the evidence to just randomized clinical trials [14]. A 2017 systematic review by Hayes et al. found a correlation between weight loss and improved physical health related QoL but did not find the same relationship with mental-health-related QoL [31]. Given that the largest increase we observed was in a component of mental health, it is possible this benefit may be unique to TRE and not weight loss. Future studies could include evaluating TRE’s simplified view of eating and its imposition of eating window structure on improving QoL.

Our finding that TRE is associated with improved QoL has significant clinical implications. Traditional caloric restriction diets are difficult for patients to maintain [32], and TRE may be a more sustainable option. As most research on TRE is relatively recent, there have not been many studies looking at its long-term sustainability. A small feasibility study of 8 adults found that at the conclusion of the 16-week TRE intervention, all individuals elected to continue TRE, and at follow-up at 36 weeks, they had maintained TRE [26]. Another study found that of 19 participants who were surveyed 16 months after the completion of their 3-month TRE intervention, 12 participants reported that they continued to do TRE at least some of the time [12]. Larger studies of TRE as well as studies directly comparing TRE and calorie restriction are necessary. It should be noted that calorie restriction has also been shown to improve some aspects of QoL, as the CALERIE 2 study by Martin et al. found improvement in general health as measured by the SF-36 [33]. They were unable to assess role limitations due to emotional health because of ceiling effects at baseline. Ceiling effects may be a problem in future QoL research as well; combining quantitative studies with qualitative studies of participants’ experiences with TRE may provide additional insight.

This study provides novel information about the effect of TRE on QoL using the SF-36 within the context of a randomized clinical trial. Strengths include the randomized trial design, weight loss achieved, monitoring intervention compliance using the mCC application, and use of the SF-36 which is the best validated QoL measurement tool. Several limitations exist. The significant overrepresentation of women in the sample is a limitation, especially because women have different SF-36 scores than men at baseline [18]. Additionally, the small sample size (*n* = 20) and short duration (12 weeks) may limit the translation of our findings.

## 5. Conclusions

The findings from this study combined with previous research indicate that in the setting of a randomized control trial, performing TRE does not adversely affect QoL and may be associated with modest improvements in QoL relative to baseline and unrestricted eating. Larger and longer studies examining the efficacy and sustainability of TRE on QOL changes and whether these changes are associated with durable long-term weight loss are warranted.

## Figures and Tables

**Table 1:** Characteristics of randomized participants. Results are presented as (mean (SD)).

	TRE (*n* = 11)	Non-TRE (*n* = 9)	*p* Value
Age, years	46.5 (12.4)	44.2 (12.3)	0.69
Sex, n, female/male	9/2	8/1	0.66
Baseline eating window	15.2 (0.7)	15.5 (1.1)	0.47
BMI	33.8 (7.6)	34.4 (7.8)	0.86

**Table 2:** Comparison of pre- and post-intervention QOL measures using ANOVA.

	TRE (*n* = 11) Mean (SD) (Range) Pre-End Intervention		Non-TRE (*n* = 9) Mean (SD) (Range) Pre-End Intervention		*p* Value for Group Effect	*p* Value for Time Effect	*p* Value for Group*Time Interaction
Physical functioning	89.1 (10.7) (70–100)	91.4 (11.6) (65–100)	83.3 (19.7) (40–100)	86.1 (8.9) (70–100)	0.32	0.27	0.91
Role limitations due to PF	88.6 (30.3) (0–100)	93.2 (22.6) (25–100)	69.4 (41.0) (0–100)	86.1 (22.0) (50–100)	0.31	0.03	0.20
Bodily pain	79.8 (15.3) (45–100)	85.5 (18.5) (45–100)	80.3 (17.1) (45–90)	81.9 (20.1) (45–100)	0.84	0.19	0.47
General health	66.4 (22.7) (5–90)	69.5 (57.5) (30–90)	66.1 (27.4) (20–100)	66.1 (25.0) (35–100)	0.86	0.58	0.58
Energy	57.7 (7.5) (45–70)	51.8 (8.1) (45–65)	52.2 (7.5) (45–70)	51.1 (8.9) (35–60)	0.27	0.15	0.32
Social function	79.5 (32.7) (0–100)	97.7 (5.1) (87.5–100)	79.2 (28.6) (12.5–100)	86.1 (21.1) (37.5–100)	0.53	0.03	0.32
Role limitations due to emotional health	66.7 (42.2) (0–100)	97.0 (10.0) (66.7–100)	63.0 (48.4) (0–100)	55.6 (44.1) (0–100)	0.15	0.21	0.04
Mental health	66.2 (8.3) (56–84)	68.7 (6.4) (60–80)	63.6 (9.3) (44–72)	64.9 (6.9) (52–72)	0.32	0.20	0.68
Health transition	52.3 (23.6) (0–100)	68.2 (4.9) (50–100)	41.7 (12.5) (25–100)	44.4 (5.6) (25–100)	0.03	0.01	0.05

**Table 3:** Post-hoc *t*-test for significant QoL measures.

	*P* Value Comparing Pre vs. Post within TRE	*P* Value Comparing Pre vs. Post within Non-TRE
Role limitations due to emotional health	0.02	0.57
Health transition	0.001	0.55

## Appendix A

**Table A1:** Correlation between weight loss and change in eating window and SF-36 measures.

	Weight Loss	Change in Eating Window
Limitations Due to Emotional Health	−0.06 (*p* = 0.87)	0.25 (*p* = 0.47)
Energy	0.02 (*p* = 0.94)	−0.15 (*p* = 0.67)
Pearson Correlation R^2^		

## Appendix B. RAND Corporation SF-36 Scoring Instructions

**Table A2:** Instructions from the SF-36 publisher on scoring each individual item on the SF-36.

Item Numbers	Change Original Response Category *	To Recoded Value of:
1, 2, 20, 22, 34, 36	1 →	100
	2 →	75
	3 →	50
	4 →	25
	5 →	0
3, 4, 5, 6, 7, 8, 9, 10, 11, 12	1 →	0
	2 →	50
	3 →	100
13, 14, 15, 16, 17, 18, 19	1 →	0
	2 →	100
21, 23, 26, 27, 30	1 →	100
	2 →	80
	3 →	60
	4 →	40
	5 →	20
	6 →	0
24, 25, 28, 29, 31	1 →	0
	2 →	20
	3 →	40
	4 →	60
	5 →	80
	6 →	100
32, 33, 35	1 →	0
	2 →	25
	3 →	50
	4 →	75
	5 →	100

* Precoded response choices as printed in the questionnaire.

**Table A3:** Instructions from the SF-36 publisher on calculating the composite scale scores from averaging individual item scores.

Scale	Number of Items	After Recoding Per Table 1, Average the Following Items
Physical functioning	10	3 4 5 6 7 8 9 10 11 12
Role limitations due to physical health	4	13 14 15 16
Role limitations due to emotional problems	3	17 18 19
Energy/fatigue	4	23 27 29 31
Emotional well-being	5	24 25 26 28 30
Social functioning	2	20 32
Pain	2	21 22
General health	5	1 33 34 35 36
